# Consensus‐based ethical best practices for performing educational point‐of‐care ultrasonography in the emergency department

**DOI:** 10.1002/aet2.10963

**Published:** 2024-03-21

**Authors:** Samantha K. Chao, Yiju T. Liu, Charles W. Kropf, Robert D. Huang, Nik Theyyunni, Lindsay A. Taylor, Janice I. Firn, Ross Kessler, Daniel R. Micheller, Alethia J. Battles, Natalja P. Rosculet, Emily E. Ager, Alyssa A. Valentyne, Christine J. Schellack, John P. Hennessy, Cameron White, Ryan V. Tucker

**Affiliations:** ^1^ Department of Emergency Medicine Michigan Medicine Ann Arbor Michigan USA; ^2^ Department of Emergency Medicine Harbor–UCLA Torrance California USA; ^3^ David Geffen School of Medicine at UCLA Los Angeles California USA; ^4^ University of Michigan Medical School Ann Arbor Michigan USA; ^5^ Department of Emergency Medicine Virginia Commonwealth University Richmond Virginia USA; ^6^ Virginia Commonwealth University School of Medicine Richmond Virginia USA; ^7^ Department of Learning Health Sciences University of Michigan Medical School Ann Arbor Michigan USA; ^8^ Center for Bioethics and Social Sciences in Medicine University of Michigan Medical School Ann Arbor Michigan USA; ^9^ Department of Emergency Medicine University of Washington Seattle Washington USA; ^10^ Michigan Medicine Ann Arbor Michigan USA; ^11^ University of Michigan College of Pharmacy Ann Arbor Michigan USA; ^12^ University of Colorado School of Medicine Aurora Colorado USA

## Abstract

**Objectives:**

There is no standardized protocol for performing educational point‐of‐care ultrasonography (POCUS) that addresses patient‐centered ethical issues such as obtaining informed consent. This study sought to define principles for ethical application of educational POCUS and develop consensus‐based best practice guidance.

**Methods:**

A questionnaire was developed by a trained ethicist after literature review with the help of a medical librarian. A diverse panel including experts in medical education, law, and bioethics; medical trainees; and individuals with no medical background was convened. The panel voted on their level of agreement with ethical principles and degree of appropriateness of behaviors in three rounds of a modified Delphi process. A high level of agreement was defined as 80% or greater consensus.

**Results:**

Panelists voted on 38 total items: 15 related to the patient consent and selection process, eight related to practices while performing educational POCUS, and 15 scenarios involving POCUS application. A high level of agreement was achieved for 13 items related to patient consent and selection, eight items related to performance practices, and 10 scenarios of POCUS application.

**Conclusions:**

Based on expert consensus, ethical best practices include obtaining informed consent before performing educational POCUS, allowing patients to decline educational POCUS, informing patients the examination is not intended to be a part of their medical evaluation and is not billed, using appropriate draping techniques, maintaining a professional environment, and disclosing incidental findings in coordination with the primary team caring for the patient. These practices could be implemented at institutions to encourage ethical use of educational POCUS when training physicians, fellows, residents, and medical students.

## INTRODUCTION

Point‐of‐care ultrasonography (POCUS) is a foundational component of the emergency medicine (EM) physician's skill set. It is a low‐cost, safe, and rapid means of obtaining detailed clinical information at the bedside that can directly impact patient care.^1^, ^2^ There is also increasing appreciation of POCUS's utility in specialties beyond EM. Ultrasound (US) training is required by the Accreditation Council for Graduate Medical Education (ACGME) for residents in EM, family medicine, and anesthesiology.^3^, ^4^, ^5^ Many large specialties, including internal medicine, pediatrics, obstetrics and gynecology, and surgery, have also advocated for integrated US curricula to improve resident competency.^6^, ^7^, ^8^, ^9^ When implementing US training at all levels of medical education, a variety of training methods are utilized, including hands‐on experiential learning. Educational POCUS is one way of providing hands‐on experience.

The American College of Emergency Physicians (ACEP) ultrasound guidelines define *educational ultrasound* as an US performed “on a patient, volunteer, or in simulation that is not intended to provide information to further the clinical care of that individual.”^1^ These US are usually performed on patients in the emergency department (ED) under the supervision of a faculty member with expertise or fellowship training in US. Educational USs are distinct from *diagnostic* USs, which are studies that are performed with the explicit goal of answering a clinical question. Additionally, diagnostic USs are typically treated as billable procedures whereas educational US studies are not.

Ethical conflicts emerge when US education involves the participation of live volunteers, especially patients actively receiving medical care in the hospital. The performance of educational POCUS highlights a common fundamental tension in medical education: trainees learn by practicing skills on patients who may not directly benefit from the examination itself. However, a greater good is served by ensuring adequate training of future independently practicing physicians.^10^ Another ethical consideration of educational POCUS is the potential discovery of abnormalities that would have otherwise gone undetected in the medical encounter—what are known as incidental findings. The prevalence of incidental findings on focused assessment with sonography in trauma examinations performed by emergency physicians is reported to be 9.4%^11^ to 26%^12^ although the sample sizes of these studies vary. In a Danish study of 405 randomly selected patients in the ED, 39.3% had a positive US finding on whole‐body POCUS.^13^ The burden of learning of an incidental finding and needing to obtain follow‐up if recommended are all potential incurred risks to the patient. Furthermore, who should deliver the news of the incidental finding and how to ensure appropriate follow‐up are challenges posed to the physicians involved.

When performing educational POCUS, given the lack of direct patient benefit and risks including encountering incidental findings, respect for patient autonomy and obtaining appropriate informed consent are imperative.^14^ Without standardization of practices, physicians and medical trainees risk compromising patient autonomy for educational benefit. Although understanding informed consent has been identified by international consensus as an essential feature of US education^15^ and best practices for communicating incidental findings in the ED have been developed,^16^ currently there is no consensus guidance on how to apply these principles in the setting of educational POCUS. The objectives of this study were to define principles for ethical application of educational bedside US and to develop related consensus‐based best practice guidance.

## METHODS

### Study design

This study utilized a modified Delphi method to build consensus. The modified Delphi technique is a well‐established educational method that uses expert opinion to achieve agreement.^17^ A literature review was performed by a trained clinical ethicist with the assistance of a medical librarian. This review was used to develop a list of relevant ethical principles and hypothetical scenarios involving the application of educational POCUS in the setting of competing ethical obligations. A structured search was performed to identify studies pertinent to our list of items (see File S1). The compiled list was adapted into a questionnaire and underwent pilot testing. The questionnaire was pilot tested with individuals who did not ultimately participate in the study, including an US fellow, two EM residents, and a layperson with legal training who had been an ED patient but who did not have medical training. There were no significant changes to the questionnaire after pilot testing. This study was reviewed by our institution's institutional review board and was determined to fall under Category 2 exemption: research involving survey procedures and the human subjects’ responses would not reasonably place them at risk.

### Study setting and population

A panel of 16 individuals with relevant expertise and experience was assembled using purposive sampling. Among the panelists were six EM attending physicians, one fellow in medical education and clinical US, four EM resident physicians, two medical students, an attorney, two clinical ethicists (one of whom was also an EM resident), and an individual with no medical background representing the patient perspective. Of the attending physicians, five were US fellowship trained, three were US fellowship directors, and one was an US division director. Two of the resident physicians were participants in an advanced US training track within their residency program. The panel included representation from multiple types of institutions and varying geographic regions. Panelist demographics are further described in Table 1. Age, race/ethnicity, and gender were self‐described. Academic practices were defined as being affiliated with a medical school and teaching hospital whereas community and county practices were defined by patient population served.^18^ Geographic regions were assigned based on the designations used by the U.S. Census Bureau.

**TABLE 1:** Demographics of the modified Delphi expert panel (*N* = 16).

Age (years)	
Younger than 20	0 (0)
20–29	3 (19)
30–39	8 (50)
40–49	4 (25)
50–59	1 (6)
60–69	0 (0)
Older than 70	0 (0)
Gender	
Women	9 (56)
Men	7 (44)
Race/ethnicity	
American Indian or Alaska Native	0 (0)
Asian	5 (31)
Black or African American	1 (6)
Native Hawaiian or other Pacific Islander	0 (0)
White	12 (75)
Hispanic or Latinx	0 (0)
Race or ethnicity not listed above	0 (0)
Practice type	
Academic	13 (81)
Community	1 (6)
County	2 (13)
Geographic region	
Midwest	13 (81)
South	1 (6)
West	2 (13)

*Note*: Data are reported as *n* (%). Responses for race/ethnicity total > 100% as respondents were allowed to select more than one category.

The research team included a trained clinical ethicist (S.C.) and an US fellowship–trained expert with experience using the modified Delphi method who assisted with data collection, methodology, and discussion moderation but did not vote (R.T.). Modified Delphi rounds were conducted electronically. Questionnaires were sent to the expert panel via Qualtrics and group discussions were held virtually using Zoom. Qualtrics was selected as the survey platform as it can track distinct information from individual respondents and store data confidentially.

### Study protocol

Three rounds of the modified Delphi process were completed, which was decided a priori. In the first round of the modified Delphi, participants were presented a questionnaire with 38 items separated into three different categories: patient selection and consent process (11 items), practices during performance of educational POCUS (14 items), and specific scenarios of educational POCUS use (13 items). In the first and second categories, participants were asked to rate how much they agree or disagree with the item on a 5‐point Likert scale (1 = strongly disagree; 2 = disagree; 3 = neutral; 4 = agree; 5 = strongly agree). In the third category, participants were asked to rate the appropriateness of the scenario described using a series of Likert‐type items (1 = extremely inappropriate; 2 = inappropriate; 3 = neither appropriate nor inappropriate; 4 = appropriate; 5 = extremely appropriate). For the purposes of the questionnaire, the term “US team” was used to represent the physicians or learners performing the educational POCUS.

After the initial voting round, the research team collated the results from Qualtrics and generated a report including each individual participant's responses, the group's responses, the group's mean score, whether the group supported or opposed an item (based on the mean score), the level of agreement for each item, and the variance in responses. This report was distributed to each participant, and the expert panel met to discuss the results of the first round. Participants were encouraged to discuss how they arrived at certain scores and to ask any clarifying questions regarding the questionnaire items. They were also allowed to suggest modifications to the items on the questionnaire, including the addition or removal of items.

Following this meeting, the questionnaire was revised and redistributed to the expert panel for a second round of voting and discussion. The same procedure was followed for the third round of the modified Delphi process.

### Key outcome measures

Level of agreement was assessed for each item on the questionnaire based on the method described by de Loe^19^ to analyze modified Delphi results. The mean score was calculated to determine which side of the scale most answers fell on. A mean of <3 indicated the group overall opposed the item. A mean of >3 indicated the group overall supported an item. A high level of agreement was reached when the number of responses on two contiguous points on either side of the 5‐point scale (1 or 2 for overall opposed items and 4 or 5 for overall supported items) reached a predetermined threshold. The group reached a high level of consensus when 80% of the responses or higher fell in the above ranges, a medium level of agreement when 70%–79.99% of responses fell in the above ranges, and a low level of agreement when <70% of responses fell in the above ranges. All items with a high level of agreement after the third round were included in the final consensus list. Items that did not achieve high agreement were excluded.

## RESULTS

### Round 1

All panelists voted in the first round. Twenty items achieved a high level of agreement, one achieved medium agreement, and 17 achieved low agreement. These results are available in Table S1 in the supplement. Based on discussion after the first round, six items were revised for clarity, including the item describing that the educational POCUS is not intended to be part of the patient's medical evaluation, specific language regarding use of “endocavitary” probes, transabdominal pelvic US, and replacing items containing “family member” with “an appropriate surrogate decision maker.” One item involving written consent was modified for content. Two items involving respect for patient personal space and using neutral language were combined into a single item covering maintenance of a professional environment. Six items describing the process for managing incidental findings were revised for content and ultimately streamlined into two items. Six items were added that addressed the following: patient consent should be generally obtained before performing educational POCUS, patients may decline the POCUS examination, patients may change their minds at any point during the POCUS, the US team will explain the process for disclosing incidental findings, a scenario involving performance of educational POCUS delaying patient disposition, and a scenario describing performing educational POCUS on a nonassenting patient without capacity who has a surrogate decision maker that could potentially consent on their behalf. Finally, two items were moved from one section to another for clarity: a patient requesting not to be informed of non–life‐threatening findings was moved from Section 2 to Section 1, and being sensitive to the patient's pain level was moved from Section 3 to Section 1.

### Round 2

All panelists voted in the second round of the modified Delphi process. The second‐round questionnaire had a total of 39 items. Thirty items achieved a high level of agreement, three achieved medium agreement, and six had low agreement. These results are available in Table S2 in the supplement. Based on discussion after this round, the item describing the US study as educational was modified for clarity. The items describing need for patient consent prior to the POCUS and only selecting patients who can consent or have surrogate decision makers present were combined into a single item to reduce redundancy. One item was added regarding preparing the patient for the number of US team members who will be present during the examination. One item regarding patients requesting to not be informed of incidental findings was removed as this is an ethically complex scenario felt to be outside of the scope of these general best practices. Finally, the item covering transvaginal pelvic US was moved from Section 2 to Section 3 for consistency.

### Round 3

All panelists voted in the third round of the modified Delphi process. The third‐round questionnaire had a total of 38 items. Thirty‐one items achieved a high level of agreement, three achieved medium agreement, and four had low agreement. The full results are available in Table S3. The consensus list of 31 items to be included in best practice guidance on performing educational POCUS is shown in Table 2. The consensus list ultimately included 13 items relevant to the patient consent and selection process, eight items relevant to practices during performance of educational POCUS, and 10 specific scenarios of US use in which the group either supported or opposed the application of educational POCUS in that scenario.

**TABLE 2:** Ethical best practice measures for performance of educational POCUS based on expert consensus.

I. Patient selection and consent process	
Informed consent should be obtained from the patient or an appropriate surrogate decision maker prior to performing educational US.	
2 Obtaining verbal consent alone is sufficient before performing educational US.	
3 Written consent may be obtained but is not necessary if verbal consent is obtained before performing educational US.	
4 Patients may decline to have an educational US performed on them.	
5 Patients may change their minds at any point and decide they no longer want an educational US performed on them.	
6 The US team should explain that the exam is educational and not intended to be a part of the patient's medical evaluation and treatment.	
7 The US team should explain that the patient will not be billed for educational US.	
8 The US team should inform the patient that declining to have an educational US will not impact their medical care.	
9 The US team should explain the risks of educational US (including discomfort, exposure on examination, incidental findings).	
10 The US team should explain that incidental findings may be encountered and will be disclosed to the patient after discussion with the primary ED team.	
11 The US team should prepare the patient for the number of US team members that will be present during the examination.	
12 The US team should give the patient an opportunity to ask questions.	
13 Consent should be obtained by one member of the US team only (as opposed to while the entire group is in the room).	
II. Practices during performance of educational US	
The US team maintains a professional environment during the exam (including respect for personal space and using neutral, anatomically accurate language).	
2 The US team uses appropriate draping techniques.	
3 Incidental findings (including threats to life or limb, findings requiring follow‐up, and pregnancy) are discussed first with the primary team.	
4 Incidental findings are communicated to the patient in a timely fashion by the provider deemed most appropriate (whether primary team or US team provider).	
5 Incidental findings are communicated to the patient in a private setting (without support persons in the room unless requested by the patient and without a large group of providers in the room).	
6 The US team encourages the patient to communicate their discomfort during the educational US.	
7 The US team is sensitive to the patient's pain level during the educational US.	
8 The US team takes into account cultural factors (religious dress, gender concordant care, etc.).	
III. Specific scenarios of US use	
The US team performs educational US on a critically ill patient who cannot consent, and this delays and interferes with care.	Oppose
2 The US team performs educational US on a non–English‐speaking patient without obtaining consent.	Oppose
3 The US team performs educational US on a non–English‐speaking patient using a trained hospital interpreter to obtain consent.	Support
4 The US team performs educational US on a patient who has already received a radiology study on the same area of interest.	Support
5 The US team performs educational US on a patient who is also a hospital employee.	Support
6 The US team performs educational US on an intoxicated patient who cannot provide consent.	Oppose
7 The US team performs an educational US on a pediatric patient who cannot consent; however, a parent provides consent.	Support
8 The US team performs educational US on an elderly patient with dementia who cannot provide consent.	Oppose
9 The US team performs educational US on a patient who does not have capacity, the appropriate surrogate decision maker provides consent, but the patient does not want the examination to be done (they do not assent to the examination).	Oppose
10 The US team performs an educational transabdominal obstetric US on a patient known to be pregnant who consents to the examination.	Support

Abbreviations: POCUS, point‐of‐care ultrasound; US, ultrasound.

## DISCUSSION

As the teaching modality of educational POCUS becomes more widespread in medical education, it is prudent to reflect on how patients are approached for these studies and how the studies themselves are performed. Currently, there are gaps in patients’ understanding of educational POCUS and trainee involvement in performing these examinations. Goldflam et al.^20^ demonstrated that a majority of surveyed patients had misconceptions regarding educational POCUS, including inaccurately thinking the study was ordered to make decisions regarding their medical care. They also showed a scripted introduction significantly improved patient understanding of the purpose and limitations of educational POCUS.^20^ Within the anesthesiology literature, there have been recent calls for increased transparency with patients regarding learner involvement in intraoperative transesophageal echocardiography.^21^, ^22^ Ivascu and Meltzer^21^ emphasize that without this transparency, physicians risk eroding the trust granted to them by patients. The existing literature suggests there is much room for improvement in terms of fully informing patients of the purposes, benefits, and risks of educational POCUS. As there is no current guidance or standardized protocol on how to approach this in the setting of educational POCUS, we identified consensus‐based best practice measures to fill this need and promote patient autonomy.

Table 2 lists all of the measures that achieved high consensus through the modified Delphi process. However, there are two items worth addressing for their ethical complexity that did not make this list. The first item describes performing transvaginal US (using an endocavitary probe) for educational purposes. In the first round of voting, the group was overall opposed to the practice with a high variability in responses and low consensus. The primary reasons for opposing educational transvaginal POCUS were related to the perceived negative impact on the patient's experience: it is uncomfortable and coercive to approach an already vulnerable population to perform this invasive examination purely for educational purposes, patients would likely be subjected to more than one invasive examination if radiology‐based pelvic US is ordered for confirmation, and the risks of discomfort are higher than other types of US because of the nature of the endocavitary probe. However, over the course of the modified Delphi process, multiple counterarguments emerged. It may be paternalistic to not offer the educational POCUS and to, therefore, not allow the patient to decide for themselves. By avoiding these studies in the educational setting, we risk not training EM residents appropriately to perform this examination, particularly those who go on to practice at institutions that rely on emergency physicians to perform pelvic POCUS for clinical decision making. Finally, we may ultimately be doing a disservice to the patient population that benefits from the information obtained from transvaginal pelvic POCUS by not giving residents the opportunity to practice them in a supervised setting. By the third round of the modified Delphi process, the group overall supported the practice of performing educational transvaginal pelvic POCUS on a patient who consents to the examination; however, there remained a high amount of variance, and the item achieved only medium consensus.

A second ethically complex item described performing educational POCUS on a patient who is intoxicated such that they do not have decision‐making capacity, but an appropriate surrogate decision maker provides consent. As a group, it was decided that patient consent must be obtained prior to performance of educational POCUS, but it remained unclear if consent provided by a surrogate decision maker was sufficient or appropriate. Throughout the three rounds, the group overall supported using the surrogate decision‐maker's consent, but variance was high and consensus was low. The primary concern was whether there was a volitional component to the patient's consent. Voluntariness is a fundamental element of informed consent and autonomous decision making.^23^ Beauchamp and Childress^23^ describe it further as “a person acts voluntarily if he or she wills the action without being under the control of another person or the control of a personal psychological condition.” In clinical practice, when a patient does not have the capacity to provide consent, we often accept the consent of a surrogate decision maker on their behalf who can speak to the preferences of the patient and the decision they would make if they had capacity in that moment. Utilizing surrogate consent makes sense in the setting of a decision that must be made, or the patient may be exposed to further harm. It does not seem to be an appropriate avenue of consent in the setting of offering an intervention like educational POCUS, which has no expected benefit to the patient and does not need to be performed. In the case of an intoxicated patient, it may be more prudent to revisit the patient when they are no longer intoxicated, have capacity, and are able to consent to or decline educational POCUS.

Furthermore, a distinction can be drawn between patients who have impaired capacity in the short term as opposed to impaired capacity in the long term. The items describing an elderly patient with dementia are an interesting contrast to the patient who is intoxicated: because dementia is a longstanding and progressive illness, these patients will likely never regain capacity. Therefore, it may be acceptable to utilize a surrogate decision maker in this setting since the approach to consenting the patient may not be different if they typically rely on a surrogate decision maker to help guide their care.

The complex and often unexpected discussion generated by these items and throughout the modified Delphi process reinforce that every patient case is unique, and there may be a variety of competing obligations and contextual factors at play. While this study presents the broad application of certain ethical principles and perspectives, the best practices recommended are not intended to be prescriptive. The resolution of ethical dilemmas involving educational scans should involve careful reflection and balancing of all relevant obligations and principles. US teachers and learners can use their judgment to decide which items to incorporate into their practice.

### LIMITATIONS

Although the expert panel has a wide range of stakeholders from different institutions and regions, experts from the Midwest and physicians were overrepresented within the group. Because of the large number of physicians in the group, discussion often skewed toward nuances of clinical practice, which may have made participation in discussion more challenging for experts from nonmedical backgrounds. In terms of questionnaire design, the items could have been improved by avoiding multibarreled items.^24^ Bias in the development of the questionnaire may also be present given the small group (two of the authors) involved in its conception; however, we attempted to mitigate this using pilot testing. Another limitation is that the questionnaire included a neutral option in the Likert‐scale responses. In the de Loe method of the modified Delphi process,^19^ it is recommended to not include a neutral option to encourage participants to take a specific stance. If experts had been forced to choose a side, this may have affected the consensus results. However, given the complex and controversial ethical issues being discussed, we felt that a neutral option would better represent the true opinions of the panelists.

## CONCLUSIONS

In conclusion, this study sought to define principles for ethical application of educational bedside point‐of‐care ultrasound and to develop a list of relevant consensus‐based best practices. Using the modified Delphi method, a diverse panel of clinicians, teachers, learners, ethicists, and community representatives achieved consensus on 31 items that represent suggested best practices for educational point‐of‐care ultrasound. We hope that utilizers of educational point‐of‐care ultrasound may incorporate these elements into their practice and reflect on the ethical complexity of educational point‐of‐care ultrasound use. We expect this research to pave the way for developing a standard way of describing educational point‐of‐care ultrasound to patients, obtaining consent for the examination, and assessing the feasibility and impact of such processes. With transparency and clear communication, we hope to meaningfully engage patient volunteers in ultrasound training and promote active partnership between patients and learners in medical education.

## CONFLICT OF INTEREST STATEMENT

The authors declare no conflicts of interest.

## Supporting information


Table S1.
