# Supplementary material for: Consensus‐based ethical best practices for performing educational point‐of‐care ultrasonography in the emergency department
Source: AEM Educ Train. 2024 Mar 21;8(2):e10963. doi: 10.1002/aet2.10963 (PMC10955606; doi:10.1002/aet2.10963)
Supplement: Supplementary file 1 — Table S1. [file AET2-8-e10963-s001.docx]

**Supplemental File 1: Literature Search Strategy**

1. PubMed search - 103 results

("education, professional"[majr] OR ((education[tiab] OR educational[tiab] OR student[tiab] OR students[tiab] OR trainee[tiab] OR trainees[tiab] OR training[tiab] OR teaching[tiab] OR clerk[tiab] OR clerkship[tiab] OR resident[tiab] OR residents[tiab] OR fellow[tiab] OR fellows[tiab] OR fellowship[tiab]) AND ("emergency medicine"[majr] OR "emergency service, hospital"[majr])))

AND

("diagnostic imaging"[sh] OR "diagnostic imaging"[majr] OR ultrasonography[majr] OR ultrasonography[ti] OR ultrasound[ti] OR radiology[majr] OR radiography[majr])

AND

("incidental findings"[majr] OR "incidental findings"[tiab] OR "unsolicited findings"[tiab] OR ethics[majr] OR ethic*[ti] OR ethics[sh] OR consent[tiab] OR "disclosure/ethics"[majr] OR "informed consent/ethics"[majr] OR "healthy volunteers"[majr])

*Search notes*

- *Mesh field tags [mesh] updated to Major Mesh [majr] for more targeted results*
- *Expanded to include other diagnostic imaging types beyond ultrasonography in case there are relevant examples there*
- *Mesh Browser is here* [*https://meshb.nlm.nih.gov/search*](https://meshb.nlm.nih.gov/search)

**Table 1S: Full List of Items and Results of Round 1 of the Delphi Process**

| Item # | Item | Mean (Variance) | Support or Oppose | Round 1 Consensus (%) |
| --- | --- | --- | --- | --- |
| **Section 1: Patient Selection and Consent Process** | | | | |
| 1 | Written consent should be obtained before performing educational ultrasound (US) | 2.25 (1.27) | Oppose | Low (68.75) |
| 2 | Obtaining verbal consent alone is sufficient before performing educational ultrasound | 4.31 (0.50) | Support | High (87.50) |
| 3 | Only patients who can provide consent or have available surrogate decision-makers are chosen to have educational US | 3.38 (1.85) | Support | Low (43.75) |
| 4 | The US team should explain that educational US is not part of the patient’s medical evaluation or treatment | 4.63 (0.25) | Support | High (100.00) |
| 5 | The US team should explain that the patient will not be billed for educational US | 4.69 (0.23) | Support | High (100.00) |
| 6 | The US team should explain the risks of educational US (including discomfort, exposure on exam, incidental findings) | 4.25 (1.00) | Support | High (93.75) |
| 7 | The US team should explain to the patient that the group performing educational US is made up of learners at different levels | 3.56 (1.60) | Support | Low (62.50) |
| 8 | The US team should inform the patient that declining to have an educational US will not impact their medical care | 4.63 (1.05) | Support | High (93.75) |
| 9 | The US team should inform the patient that images of the educational US will be saved securely and may be used later for quality improvement and teaching purposes | 3.94 (1.40) | Support | Medium (75.00) |
| 10 | The US team should give the patient an opportunity to ask questions | 4.88 (0.12) | Support | High (100.00) |
| 11 | Consent should be obtained by one member of the US team only (as opposed to while the entire group is in the room) | 3.81 (0.83) | Support | Low (62.50) |
| **Section 2: Practices During Performance of Educational Ultrasound** | | | | |
| 12 | The US team maintains respect for the patient’s personal space | 4.44 (1.20) | Support | High (87.50) |
| 13 | The US team uses appropriate draping techniques | 4.63 (0.25) | Support | High (100.00) |
| 14 | The US team uses neutral and anatomically accurate language | 4.56 (0.40) | Support | High (93.75) |
| 15 | Incidental findings that are threats to life or limb are communicated immediately to the patient | 3.88 (1.45) | Support | Low (68.75) |
| 16 | Incidental findings that are threats to life or limb are communicated immediately to the primary care team | 4.81 (0.16) | Support | High (100.00) |
| 17 | Incidental findings that are non-life threatening but require close follow-up are communicated to the patient | 3.50 (2.27) | Support | Low (56.25) |
| 18 | Incidental findings that are non-life threatening but require close follow-up are communicated to the primary care team | 4.75 (0.20) | Support | High (100.00) |
| 19 | An incidental finding of pregnancy is communicated to the patient | 4.13 (1.45) | Support | Low (68.75) |
| 20 | An incidental finding of pregnancy is communicated to the primary care team | 4.75 (0.20) | Support | High (100.00) |
| 21 | Any incidental finding, regardless of level of concern it presents, is communicated to the patient privately | 3.94 (1.80) | Support | Low (68.75) |
| 22 | A patient requests to not be informed of non-life threatening incidental findings, and the US team honors the request | 4.13 (0.65) | Support | High (87.50) |
| 23 | The US team performs a transvaginal pelvic examination as an educational US (using an intracavitary probe) | 2.88 (1.72) | Oppose | Low (37.50) |
| 24 | The US team encourages the patient to communicate their discomfort during the educational US | 4.75 (0.20) | Support | High (100.00) |
| 25 | The US team takes into account cultural factors (religious dress, gender concordant care, etc.) | 4.63 (0.38) | Support | High (93.75) |
| **Section 3: Specific Scenarios of Educational Ultrasound Use** | | | | |
| 26 | The US team performs educational US on a critically ill patient who cannot consent, and this delays and interferes with care | 1.69 (1.03) | Oppose | High (87.50) |
| 27 | The US team performs educational US on a critically ill patient who cannot consent, and it does not delay or interfere with care | 2.81 (1.50) | Oppose | Low (50.00) |
| 28 | The US team performs educational US on a non-English speaking patient without obtaining consent | 1.38 (0.38) | Oppose | High (93.75) |
| 29 | The US team performs educational US on a non-English speaking patient, a family member provides consent in English | 2.44 (1.06) | Oppose | Low (43.75) |
| 30 | The US team performs educational US on a patient who has already received a radiology study on the same area of interest | 3.63 (1.32) | Support | Low (56.25) |
| 31 | The US team performs educational US on a patient who has abdominal pain, and the scan increases abdominal pain | 3.13 (0.92) | Support | Low (25.00) |
| 32 | The US team performs educational US on a patient who is also a hospital employee | 3.75 (0.73) | Support | Low (62.50) |
| 33 | The US team performs educational US on an intoxicated patient who cannot provide consent | 1.56 (0.40) | Oppose | High (93.75) |
| 34 | The US team performs educational US on an intoxicated patient who cannot consent; however, a family member is present to provide consent | 3.06 (1.93) | Support | Low (43.75) |
| 35 | The US team performs an educational US on a pediatric patient who cannot consent; however, a parent is present to provide consent | 4.06 (0.73) | Support | High (81.25) |
| 36 | The US team performs educational US on an elderly patient with dementia who cannot provide consent | 1.81 (0.83) | Oppose | High (81.25) |
| 37 | The US team performs educational US on an elderly patient with dementia who cannot provide consent; however, a family member is present to provide consent | 3.38 (1.32) | Support | Low (50.00) |
| 38 | The US team performs an educational pelvic US on a patient known to be pregnant | 3.31 (1.70) | Support | Low (37.50) |

US = Ultrasound.

**Table 2S: Results of Round 2 of the Delphi Process**

| Item # | Item | Mean (Variance) | Support or Oppose | Round 2 Consensus (%) |  |  |  |  |
| --- | --- | --- | --- | --- | --- | --- | --- | --- |
| **Section 1: Patient Selection and Consent Process** | | | | |  |  |  |  |
| 1 | *Patient consent should be obtained prior to performing an educational US* | 4.81 (0.16) | Support | High (100.00) |  |  |  |  |
| 2 | Obtaining verbal consent alone is sufficient before performing educational US | 4.50 (0.67) | Support | High (93.75) |  |  |  |  |
| 3 | *Written consent may be obtained but is not necessary if verbal consent is obtained before performing educational US* | 4.50 (0.53) | Support | High (87.50) |  |  |  |  |
| 4 | *Patients may decline to have an educational US performed on them* | 4.88 (0.12) | Support | High (100.00) |  |  |  |  |
| 5 | *Patients may change their minds at any point and decide they no longer want an educational US performed on them* | 4.88 (0.12) | Support | High (100.00) |  |  |  |  |
| 6 | Only patients who can provide consent or have available surrogate decision-makers are chosen to have educational US | 4.38 (0.65) | Support | High (93.75) |  |  |  |  |
| 7 | The US team should explain that educational US is not *intended to be a part* of the patient’s medical evaluation and treatment | 4.69 (0.23) | Support | High (100.00) |  |  |  |  |
| 8 | The US team should explain that the patient will not be billed for educational US | 4.56 (0.53) | Support | High (87.50) |  |  |  |  |
| 9 | The US team should inform the patient that declining to have an educational US will not impact their medical care | 4.56 (1.60) | Support | High (93.75) |  |  |  |  |
| 10 | The US team should explain the risks of educational US (including discomfort, exposure on exam, incidental findings) | 4.75 (0.20) | Support | High (100.00) |  |  |  |  |
| 11 | *The US team should explain that significant incidental findings may be encountered and will be disclosed to the patient after discussion with the primary team* | 4.63 (0.25) | Support | High (100.00) |  |  |  |  |
| 12 | *The patient can request to not be informed of non life-threatening incidental findings* | 4.38 (0.52) | Support | High (87.50) |  |  |  |  |
| 13 | The US team should explain to the patient that the group performing educational US is made up of learners | 4.06 (1.00) | Support | Low (68.75) |  |  |  |  |
| 14 | The US team should inform the patient that educational US images will be saved securely if that is done at their institution | 3.63 (1.85) | Support | Low (62.50) |  |  |  |  |
| 15 | The US team should give the patient an opportunity to ask questions | 4.88 (0.12) | Support | High (100.00) |  |  |  |  |
| 16 | Consent should be obtained by one member of the US team only (as opposed to while the entire group is in the room) | 4.13 (0.65) | Support | Medium (75.00) |  |  |  |  |
| **Section 2: Practices During Performance of Educational Ultrasound** | | | | |  |  |  |  |
| 17 | *The US team maintains a professional environment during the exam (including respect for personal space and using neutral, anatomically accurate language)* | 4.88 (0.12) | Support | High (100.00) |  |  |  |  |
| 18 | The US team uses appropriate draping techniques | 4.88 (0.12) | Support | High (100.00) |  |  |  |  |
| 19 | *Incidental findings (including threats to life or limb, findings requiring follow-up, and pregnancy) are discussed first with the primary team* | 4.13 (1.32) | Support | High (87.50) |  |  |  |  |
| 20 | *Incidental findings are communicated to the patient in a timely fashion by the provider deemed most appropriate (whether primary team or US team provider)* | 4.69 (0.23) | Support | High (100.00) |  |  |  |  |
| 21 | *Incidental findings are communicated to the patient in a private setting (without support persons in the room unless requested by the patient and without a large group of providers in the room)* | 4.25 (0.60) | Support | High (81.25) |  |  |  |  |
| 22 | The US team performs a transvaginal pelvic examination as an educational (using an *endocavitary* probe) | 2.75 (1.80) | Oppose | Low (43.75) |  |  |  |  |
| 23 | The US team encourages the patient to communicate their discomfort during the educational US | 4.69 (0.36) | Support | High (93.75) |  |  |  |  |
| 24 | *The US team is sensitive to the patient’s pain level during educational US* | 4.69 (0.36) | Support | High (93.75) |  |  |  |  |
| 25 | The US team takes into account cultural factors (religious dress, gender concordant care, etc.) | 4.94 (0.06) | Support | High (100.00) |  |  |  |  |
| **Section 3: Specific Scenarios of Educational Ultrasound Use** | | | | |  |  |  |  |
| 26 | The US team performs educational US on a critically ill patient who cannot consent, and this delays and interferes with care | 1.06 (0.06) | Oppose | High (100.00) |  |  |  |  |
| 27 | The US team performs educational US on a critically ill patient who cannot consent, and it does not delay or interfere with care | 2.50 (1.20) | Oppose | Low (56.25) |  |  |  |  |
| 28 | *The US team performs educational US on a consenting patient, but the scan delays the patient’s disposition (i.e., discharge or moving to an inpatient bed)* | 2.50 (1.33) | Oppose | Low (62.50) |  |  |  |  |
| 29 | The US team performs educational US on a non-English speaking patient without obtaining consent | 1.25 (0.20) | Oppose | High (100.00) |  |  |  |  |
| 30 | The US team performs educational US on a non-English speaking patient *using a trained hospital interpreter to obtain consent* | 4.69 (0.23) | Support | High (100.00) |  |  |  |  |
| 31 | The US team performs educational US on a patient who has already received a radiology study on the same area of interest | 3.94 (0.46) | Support | Medium (75.00) |  |  |  |  |
| 32 | The US team performs educational US on a patient who is also a hospital employee | 4.00 (0.53) | Support | Medium (75.00) |  |  |  |  |
| 33 | The US team performs educational US on an intoxicated patient who cannot provide consent | 1.44 (0.26) | Oppose | High (100.00) |  |  |  |  |
| 34 | The US team performs educational US on an intoxicated patient who cannot consent; however, *an appropriate surrogate decision-maker* provides consent | 3.63 (1.18) | Support | Low (56.25) |  |  |  |  |
| 35 | The US team performs an educational US on a pediatric patient who cannot consent; however, a parent provides consent | 4.31 (0.50) | Support | High (87.50) |  |  |  |  |
| 36 | The US team performs educational US on an elderly patient with dementia who cannot provide consent | 1.56 (0.80) | Oppose | High (87.50) |  |  |  |  |
| 37 | The US team performs educational US on an elderly patient with dementia who cannot provide consent; however, an *appropriate surrogate decision-maker* provides consent | 4.00 (1.20) | Support | High (87.50) |  |  |  |  |
| 38 | *The US team performs an educational US on a patient who does not have capacity, the appropriate surrogate decision-maker provides consent, but the patient does not want the exam to be done (they do not assent to the exam)* | 1.69 (0.63) | Oppose | High (81.25) |  |  |  |  |
| 39 | The US team performs an educational transabdominal obstetric US on a patient known to be pregnant *who consents to the exam* | 4.31 (0.50) | Support | High (87.50) |  |  |  |  |

US = Ultrasound. Italicized items are revised from prior round.

**Table 3S: Results of Round 3 of the Delphi Process**

| Item # | Item | Mean (Variance) | Support or Oppose | Round 3 Consensus (%) |
| --- | --- | --- | --- | --- |
| **Section 1: Patient Selection and Consent Process** | | | | |
| 1 | *Informed consent should be obtained from the patient or an appropriate surrogate decision-maker prior to performing educational US* | 5.00 (0.00) | Support | High (100.00) |
| 2 | Obtaining verbal consent alone is sufficient before performing educational US | 4.69 (0.63) | Support | High (93.75) |
| 3 | Written consent may be obtained but is not necessary if verbal consent is obtained before performing educational US | 4.69 (0.36) | Support | High (93.75) |
| 4 | Patients may decline to have an educational US performed on them | 5.00 (0.00) | Support | High (100.00) |
| 5 | Patients may change their minds at any point and decide they no longer want an educational US performed on them | 5.00 (0.00) | Support | High (100.00) |
| 6 | The US team should explain that educational US is not intended to be a part of the patient’s medical evaluation and treatment | 4.81 (0.16) | Support | High (100.00) |
| 7 | The US team should explain that the patient will not be billed for educational US | 4.38 (0.92) | Support | High (81.25) |
| 8 | The US team should inform the patient that declining to have an educational US will not impact their medical care | 4.94 (0.06) | Support | High (100.00) |
| 9 | The US team should explain the risks of educational US (including discomfort, exposure on exam, incidental findings) | 4.44 (1.60) | Support | High (81.25) |
| 10 | The US team should explain that *incidental findings* may be encountered and will be disclosed to the patient after discussion with the primary team | 4.25 (1.27) | Support | High (81.25) |
| 11 | The US team should explain to the patient that the group performing educational US is made up of learners | 3.75 (1.67) | Support | Low (62.50) |
| 12 | *The US team should prepare the patient for the number of US team members that will be present during the exam* | 4.31 (0.63) | Support | High (81.25) |
| 13 | The US team should inform the patient that educational US images will be saved securely if that is done at their institution | 3.19 (1.90) | Support | Low (43.75) |
| 14 | The US team should give the patient an opportunity to ask questions | 5.00 (0.00) | Support | High (100.00) |
| 15 | Consent should be obtained by one member of the US team only (as opposed to while the entire group is in the room) | 4.31 (0.50) | Support | High (87.50) |
| **Section 2: Practices During Performance of Educational Ultrasound** | | | | |
| 16 | The US team maintains a professional environment during the exam (including respect for personal space and using neutral, anatomically accurate language) | 4.94 (0.06) | Support | High (100.00) |
| 17 | The US team uses appropriate draping techniques | 4.94 (0.06) | Support | High (100.00) |
| 18 | Incidental findings (including threats to life or limb, findings requiring follow-up, and pregnancy) are discussed first with the primary team | 4.38 (1.05) | Support | High (93.75) |
| 19 | Incidental findings are communicated to the patient in a timely fashion by the provider deemed most appropriate (whether primary team or US team provider) | 4.81 (0.16) | Support | High (100.00) |
| 20 | Incidental findings are communicated to the patient in a private setting (without support persons in the room unless requested by the patient and without a large group of providers in the room) | 4.44 (0.40) | Support | High (93.75) |
| 21 | The US team encourages the patient to communicate their discomfort during the educational US | 4.88 (0.120 | Support | High (100.00) |
| 22 | The US team is sensitive to the patient’s pain level during educational US | 4.88 (0.12) | Support | High (100.00) |
| 23 | The US team takes into account cultural factors (religious dress, gender concordant care, etc.) | 4.94 (0.06) | Support | High (100.00) |
| **Section 3: Specific Scenarios of Educational Ultrasound Use** | | | | |
| 24 | The US team performs educational US on a critically ill patient who cannot consent, and this delays and interferes with care | 1.06 (0.06) | Oppose | High (100.00) |
| 25 | The US team performs educational US on a critically ill patient who cannot consent, and it does not delay or interfere with care | 2.00 (1.33) | Oppose | Medium (75.00) |
| 26 | The US team performs educational US on a consenting patient, but the scan delays the patient’s disposition (i.e., discharge or moving to an inpatient bed) | 2.88 (1.32) | Oppose | Low (43.75) |
| 27 | The US team performs educational US on a non-English speaking patient without obtaining consent | 1.06 (0.06) | Oppose | High (100.00) |
| 28 | The US team performs educational US on a non-English speaking patient using a trained hospital interpreter to obtain consent | 4.81 (0.16) | Support | High (100.00) |
| 29 | The US team performs educational US on a patient who has already received a radiology study on the same area of interest | 4.31 (0.50) | Support | High (87.50) |
| 30 | The US team performs educational US on a patient who is also a hospital employee | 4.44 (0.53) | Support | High (87.50) |
| 31 | The US team performs educational US on an intoxicated patient who cannot provide consent | 1.25 (0.33) | Oppose | High (93.75) |
| 32 | The US team performs educational US on an intoxicated patient who cannot consent; however, an appropriate surrogate decision-maker provides consent | 3.25 (1.93) | Support | Low (56.25) |
| 33 | The US team performs an educational US on a pediatric patient who cannot consent; however, a parent provides consent | 4.38 (0.38) | Support | High (93.75) |
| 34 | The US team performs educational US on an elderly patient with dementia who cannot provide consent | 1.19 (0.30) | Oppose | High (93.75) |
| 35 | The US team performs educational US on an elderly patient with dementia who cannot provide consent; however, an appropriate surrogate decision-maker provides consent | 3.94 (1.13) | Support | Medium (75.00) |
| 36 | The US team performs an educational US on a patient who does not have capacity, the appropriate surrogate decision-maker provides consent, but the patient does not want the exam to be done (they do not assent to the exam) | 1.19 (0.16) | Oppose | High (100.00) |
| 37 | The US team performs an educational transabdominal obstetric US on a patient known to be pregnant who consents to the exam | 4.69 (0.36) | Support | High (93.75) |
| 38 | *The US team performs an educational transvaginal pelvic US (using an endocavitary probe) on a patient who consents to the exam* | 4.19 (1.23) | Support | Medium (75.00) |

US = Ultrasound. Italicized items are revised from prior round.
